# Gone Hence

**Published:** 1882-02

**Authors:** 


### GONE HENCE.

DIED:—January 16, 1882, in his 87th year, Lyman Taft, *our Father*. His period of life had much extended beyond the usual allotment of men. He passed away because the machinery of life had exhausted its resources—ran down—and his spirit was prepared for a new and better state of existence. These transitions are constantly taking place, and happy, yea, thrice happy is he who is ready when the call comes.

